# Integrating digital cultural detective games with social emotional learning to foster cultural sensitivity and intercultural empathy among kindergarten teachers: a mixed-methods study

**DOI:** 10.3389/fpsyg.2025.1717522

**Published:** 2026-01-15

**Authors:** Guan Zheng Chen, Yuying Fan, Zhongyuan Liao

**Affiliations:** 1 School of Foreign Languages, Guangzhou Xinhua University, Guangzhou, China; 2 School of Public Administration, Guangzhou Xinhua University, Guangzhou, China; 3 School of Teacher Education, Hezhou University, Hezhou, China

**Keywords:** cultural sensitivity, digital cultural detective game, game-based learning, intercultural empathy, kindergarten teachers, social–emotional learning

## Abstract

**Background:**

Developing cultural sensitivity and intercultural empathy is essential for kindergarten teachers working in increasingly diverse and globalized environments, particularly those in bilingual or international kindergartens. Traditional cultural training programs may not effectively cultivate these skills. This study explores the potential of integrating digital cultural detective games into social–emotional learning (SEL) to enhance cultural sensitivity and intercultural empathy among bilingual kindergarten teachers.

**Methods:**

A mixed research method was employed, combining a randomized controlled trial (RCT) with a phenomenological approach. Sixty bilingual kindergarten teachers from a city in China were recruited and randomly assigned to an experimental group with a training program incorporating digital cultural detective games, or a control group with traditional cultural training. Participants completed a questionnaire to test cultural sensitivity and intercultural empathy before and after the intervention. Besides, they were also assessed using the intercultural empathy observation checklist and the cultural sensitivity observation checklist. Finally, semi-structured interviews were conducted to explore their experiences and perceptions.

**Results:**

Quantitative findings revealed significant improvements in cultural sensitivity and intercultural empathy within the experimental group compared to the control group. Qualitative findings identified five themes, demonstrating the impact of different intervention measures on participants’ cultural sensitivity and intercultural empathy, as well as the expected benefits and limitations of each measure.

**Conclusion:**

This study demonstrates that incorporating digital cultural detective games into SEL can effectively improve cultural sensitivity and intercultural empathy among bilingual kindergarten teachers in China. The findings highlight the potential of innovative digital games in cultural training programs to promote essential social and emotional competencies and intercultural communication skills. Future research is recommended to investigate the long-term effects and applicability of this approach in various educational contexts and with different populations.

## 1 Introduction

In today’s increasingly interconnected and globalized world, fostering cultural sensitivity and intercultural empathy among educators has become paramount. Drawing on Gay (2018) and Ladson-Billings (2014) theories, teachers’ cultural sensitivity directly affects the development of children’s cultural awareness, empathy, and inclusive behavior. Kindergarten teachers, in particular, play a pivotal role in shaping the early experiences and identities of children (Chen and Fan, 2025a; Chen and Fan, 2025b). Children begin to internalize cultural differences as early as age three, making the preschool years particularly sensitive for cultivating inclusive attitudes (Aboud, 2008). Unlike educators working with older students who may have already formed cultural biases, kindergarten teachers have the unique opportunity to shape children’s cultural perceptions during the most malleable developmental period (Banks, 2008). Driven by globalization and the growing demand for international education, bilingual kindergartens in China, offering instruction in both Chinese and English, are rapidly developing. These kindergartens bring together children from diverse linguistic and cultural backgrounds, presenting teachers with unique opportunities and challenges.

To create inclusive and equitable learning environments, educators must be equipped with the skills and knowledge necessary to understand, respect, and appreciate cultural differences (Anderton and King, 2016). According to the Collaborative for Academic, Social, and Emotional Learning (CASEL), social and emotional learning (SEL) not only promotes students’ mental health but also enhances their academic performance and interpersonal relationships (Collaborative for Academic, Social, and Emotional Learning, 2021). SEL encompasses essential competencies such as self-awareness, self-management, social awareness, relationship skills, and responsible decision-making, which are critical for building a supportive educational environment conducive to effective learning (Chen and Fan, 2025a; Chen and Fan, 2025b; Davis, 1983; Stott and Neustaedter, 2013).

In recent years, game-based teaching and learning have emerged as powerful tools for education and social change (Gee, 2003; Yang and Kang, 2022). Previous studies have demonstrated that game-based learning can significantly enhance educational outcomes across various contexts, underscoring its potential as a valuable tool in teacher training programs (Chen and Fan, 2025a; Chen and Fan, 2025b; Squire, 2013). An expanding body of literature on games, which are intentionally designed to achieve educational, political, or social objectives, has demonstrated their potential for promoting critical thinking, debate, and active learning (McGonigal, 2011). For example, the game “PeaceMaker” has successfully altered students’ perceptions and attitudes toward the Israeli-Palestinian conflict, showcasing how digital engagement can lead to greater understanding of complex social issues (Alhabash and Wise, 2012). Additionally, Frasca (2001) proposed that video games serve as effective mediums for fostering critical discussions, thus enhancing players’ engagement with intricate societal challenges. However, there was insufficient attention given to how these digital games can be tailored to facilitate intercultural communication and understanding among educators.

Despite the encouraging impacts of games, significant research gaps remain concerning their integration within SEL frameworks to effectively promote cultural sensitivity and empathy among educators. A notable gap identified in existing literature is the lack of integration of cultural detective methods within digital platforms such as Gather.town to create immersive cultural learning experiences. This innovative approach could leverage the platform’s unique features, including its immersive, interactive, and collaborative capabilities, to facilitate an engaging exploration of cultural diversity and enhance intercultural understanding among kindergarten teachers (Zhao and McClure, 2024). By embedding SEL competencies within the activities of these digital cultural detective games, there exists a great opportunity to not only foster experiential learning but also to cultivate deeper empathy for diverse perspectives (Gatti et al., 2019).

This study proposes an integrated theoretical framework that synthesizes SEL framework, digital game-based learning theory, and intercultural communication competence theory. The convergence of these theories yields distinctive insights into the potential of digital cultural detective games in enhancing cultural sensitivity and intercultural empathy among kindergarten teachers within a Chinese context. Existing literature often overlooks the practical implications of such theoretical integrations for teaching methodologies, emphasizing a pressing need for comprehensive frameworks that can guide educators in adopting these innovative pedagogical approaches (Hirumi et al., 2010; Kapp, 2012).

Highlighting the importance of these frameworks, it is clear that further research is essential to validate the effectiveness of digital cultural detective games within SEL contexts. By addressing these research gaps, this study aims to enrich the scholarly discourse surrounding games and provide evidence-based recommendations for educational institutions and teacher training programs that aspire to integrate game-based learning strategies to enhance cultural sensitivity and intercultural empathy within the Chinese bilingual kindergarten environment. Three research questions have emerged from this study design, each aimed at elucidating the potential benefits and challenges associated with the integration of digital cultural detective games and SEL frameworks for bilingual kindergarten teachers in cultural training programs.

Research Questions:

- RQ1: Does the integration of digital cultural detective games and social emotional learning significantly enhance cultural sensitivity among bilingual kindergarten teachers in a cultural training program compared to a control group without the games?
- RQ2: Does the integration of digital cultural detective games and social emotional learning significantly enhance intercultural empathy among bilingual kindergarten teachers in a cultural training program compared to a control group without the games?
- RQ3: How do bilingual kindergarten teachers perceive the impact of participating in digital cultural detective games integrated with social emotional learning in a cultural training program on their cultural sensitivity and intercultural empathy?

## 2 Literature review

### 2.1 Cultural sensitivity and intercultural empathy

Cultural sensitivity is defined as the recognition and respect for cultural differences, coupled with the ability to interact effectively with individuals from varied backgrounds (Foronda, 2008). When it comes to empathy, it is influenced by different traditional values. Intercultural empathy means placing oneself into the cultural background of the target language and being able to effectively communicate the understanding of that world (Davis, 1983; Zhu, 2011). Cultural detective methods refer to a set of tools and strategies designed to enhance intercultural competence by promoting understanding and appreciation across diverse cultural contexts (Gabriel, 2016; Zhao and McClure, 2024). These methods typically involve engaging participants in role-playing, storytelling, and problem-solving activities that help identify cultural biases and develop empathy for others’ perspectives (Anderton and King, 2016). While existing studies emphasize the importance of cultural sensitivity in promoting students’ understanding and acceptance of diversity, the specific implementation of cultural sensitivity in teacher education and training remains underexplored, particularly in the context of Chinese education. Thus, there is a pressing need to thoroughly investigate how educators can implement cultural sensitivity strategies in the classroom.

### 2.2 Definition and importance of the social and emotional learning framework

Collaborative for academic, social, and emotional learning framework has shown significant relevance in educational reforms, providing educators with essential skills and tools to foster understanding and inclusivity among students from various cultural backgrounds. It defines SEL as an evidence-based approach to teaching and learning that emphasizes the development of five core competencies: self-awareness, self-management, social awareness, relationship skills, and responsible decision-making. These competencies are crucial in cultivating well-rounded individuals capable of navigating diverse cultural contexts (Collaborative for Academic, Social, and Emotional Learning, 2021). Research has shown that the SEL framework effectively promotes positive student outcomes, including improved academic achievement, enhanced social skills, and reduced behavioral problems (Yang and Kang, 2022). As globalization accelerates, the need for cultural adaptability and cross-cultural communication skills has become increasingly important. However, although literature highlights the importance of SEL in student development, there are few empirical studies addressing how teachers can specifically apply SEL in the classroom to enhance cultural sensitivity and intercultural empathy.

### 2.3 Role of game-based learning in education

In recent years, game-based learning has garnered substantial interest from researchers and educators, with an increasing body of literature recognizing the potential of games as powerful teaching tools (Bogost, 2011; Chen and Fan, 2025a; Chen and Fan, 2025b; Frasca, 2001; McGonigal, 2011; Ruggiero, 2014). Research indicates that games can cultivate critical thinking, problem-solving skills, collaboration, and experiential learning, while also providing new platforms for situated learning and identity development (Greenhalgh, 2016; Gee, 2003; Hirumi et al., 2010; Prensky, 2003; Yang and Kang, 2022). Despite the recognized potential of game-based learning, there is still a gap in research focusing on how to effectively integrate games within the SEL framework to enhance teachers’ cultural sensitivity and empathy.

### 2.4 Utilization of the Gather.town platform

Gather.town is a web-based virtual environment that allows users to interact in real time through customizable avatars and spatial audio. The platform offers features such as video conferencing, screen sharing, and collaborative whiteboards, making it highly suitable for conducting engaging and immersive digital games (Zhao and McClure, 2024). The research of Chen et al. (2025) has proven that Gather.town can help improve students’ foreign language reading abilities. Moreover, Chen and Fan (2025a) and Chen and Fan (2025b) emphasized the effectiveness of the HyFlex learning model and Gather.town in improving the cross-cultural competence of kindergarten teachers, highlighting the critical role technology can play in supporting educators as they navigate multicultural environments. Integrating cultural detective methods into the Gather.town platform to create digital cultural detective games, with the opportunity to utilize the platform’s unique features to cultivate participants’ cultural sensitivity and cross-cultural empathy.

Based on the above, this study attempts to provide a comprehensive framework by integrating the SEL framework, game-based learning, and Gather.town platform to create a more inclusive and culturally sensitive educational environment, and enhance the cultural sensitivity and intercultural empathy of bilingual kindergarten teachers.

## 3 Method

### 3.1 Participants

Recruitment was conducted in two phases. In the first phase, purposive sampling was used to identify eligible institutions. The selection criteria were as follows: the kindergarten offered bilingual instruction in Chinese and English; the kindergarten teachers had at least 1 year of experience teaching to a multicultural student body; the kindergarten allowed teachers to participate in an eight-week training program; the kindergarten had the technological infrastructure to use the Gather.town platform. Ultimately, 8 kindergartens met all the criteria and agreed to participate in the study. In the second phase, a simple random sampling method was used to recruit teachers. Sixty participants were randomly selected from 120 qualified bilingual kindergarten teachers across the 8 kindergartens using a computer-generated random number table. These 60 participants were then randomly assigned to either the experimental group (*n* = 30) or the control group (*n* = 30) using block randomization to ensure a balanced number of participants in both groups. The demographic information of the participants revealed a gender distribution of 27 females (90%) and 3 males (10%) in each group. The participants’ ages ranged from 22 to 45 years old. In terms of educational background, both groups consisted of 24 participants with bachelor’s degrees (80%) and 6 participants with master’s degrees (20%).

### 3.2 Research design

During the eight-week training, there were a weekly teaching session on a specific theme, such as American culture, Japanese culture and Indian culture. These cultures represent the most common national backgrounds of expatriate families enrolled in participating in bilingual kindergartens. By focusing training on cultures participants would likely encounter professionally, the program maximized practical applicability and motivated engagement. In addition, the selected cultures cover six continents and represent major regions of the world: North America (United States), East Asia (Japan, China), South Asia (India), South America (Brazil), Africa (South Africa), Eurasia (Russia), and Oceania (Australia). Details can be seen in Appendix A. An innovative and interactive model for the experimental group and a traditional lecture-based format for the control group.

For the experimental group, the intervention incorporated digital cultural detective games, SEL principles, and the Gather.town platform. At the start of each session, the instructor introduced the theme and objectives, detailing how the digital cultural detective games facilitate exploration of various cultures. Bilingual kindergarten teachers worked as “cultural detectives” in small groups within the Gather.town virtual environment, solving puzzles and navigating challenges related to diverse cultures. Each group member had designated roles, fostering collaboration and ensuring active participation. As they engage in treasure hunt activities, instructors circulated among the groups, providing immediate guidance and support, encouraging critical thinking, and prompting bilingual kindergarten teachers to collaboratively discuss and analyze cultural clues.

In contrast, the control group engaged in a traditional cross-cultural course that emphasizes lectures, reading assignments, and comprehension questions over the same eight-week timeframe. Instructors provided detailed lectures each week on fundamental aspects of the cultures being studied, such as history, customs, and societal norms. Bilingual kindergarten teachers passively received this information, which serves as the foundation for subsequent group discussions. Reading materials, including articles and case studies, are assigned to deepen bilingual kindergarten teachers’ understanding of cultural nuances, allowing them to explore topics at a more granular level. However, the learning process in this group was structured primarily around individual assessments. Instructors designed comprehension questions to ensure bilingual kindergarten teachers grasp the content discussed in lectures and readings. The focus was largely on recitation and understanding of facts rather than on active engagement with the material.

While both groups covered similar cultural topics, their learning model totally differed. The experimental group thrived in an interactive setting emphasizing collaboration and critical thinking. The instructors’ role was to facilitate discovery and provide timely support, making learning dynamic and student-centered. By contrast, the control group prioritized acquiring knowledge through lectures and texts.

#### 3.2.1 Instructional materials for the experimental group

##### 3.2.1.1 Digital cultural detective games

The digital cultural detective games served as a key instructional tool for the experimental group, promoting bilingual kindergarten teachers’ cultural knowledge through interactivity and engagement. At the beginning of each class, the instructor introduced the game’s objectives and explained how it facilitates bilingual kindergarten teachers’ learning about different cultures. The instructor outlined the basic rules and provided relevant cultural background information to guide bilingual kindergarten teachers’ understanding of the cultural themes. Bilingual kindergarten teachers were then divided into small groups to participate in the game on the Gather.town platform, with each group member assigned different roles to foster collaborative learning. The instructor circulated during gameplay, observed interactions, and provided necessary guidance.

During the game, bilingual kindergarten teachers took on the role of “cultural detectives,” exploring and solving puzzles based on provided clues. They applied critical thinking to analyze the clues and engage in discussions with peers, sharing varying insights and interpretations. Bilingual kindergarten teachers also used the LESCANT Model Worksheet to record their observations and reflections, assisting them in systematically analyzing various cultural dimensions (Victor, 1992). After gameplay, the instructor facilitated a reflection discussion, encouraging bilingual kindergarten teachers to share their findings which enhance their understanding of cultural knowledge.

##### 3.2.1.2 LESCANT model worksheet

This worksheet was a crucial tool that guides bilingual kindergarten teachers in the experimental group as they explore various cultural dimensions, specifically language, environment, social organization, context, authority, nonverbal communication, and time. Each week, bilingual kindergarten teachers engaged in new culturally themed treasure hunt activities on the Gather.town platform, focusing on the cultures and traditions of different nations. These activities aimed to develop critical thinking skills, cultural understanding, and empathy by incorporating cultural clues into the treasure hunts and puzzles.

The worksheet provided overviews for each cultural dimension, along with guiding questions and sections for note-taking. Details can be seen in Appendix G. As bilingual kindergarten teachers participated in the treasure hunts, they documented their observations and insights, facilitating deeper explorations of cultural concepts. The structured format of the LESCANT Model Worksheet (Victor, 1992) not only supported bilingual kindergarten teachers in systematically analyzing different cultural dimensions but also encouraged group discussions and reflective debriefing sessions afterward.

##### 3.2.1.3 Social emotional learning principles

**Figure 1:**
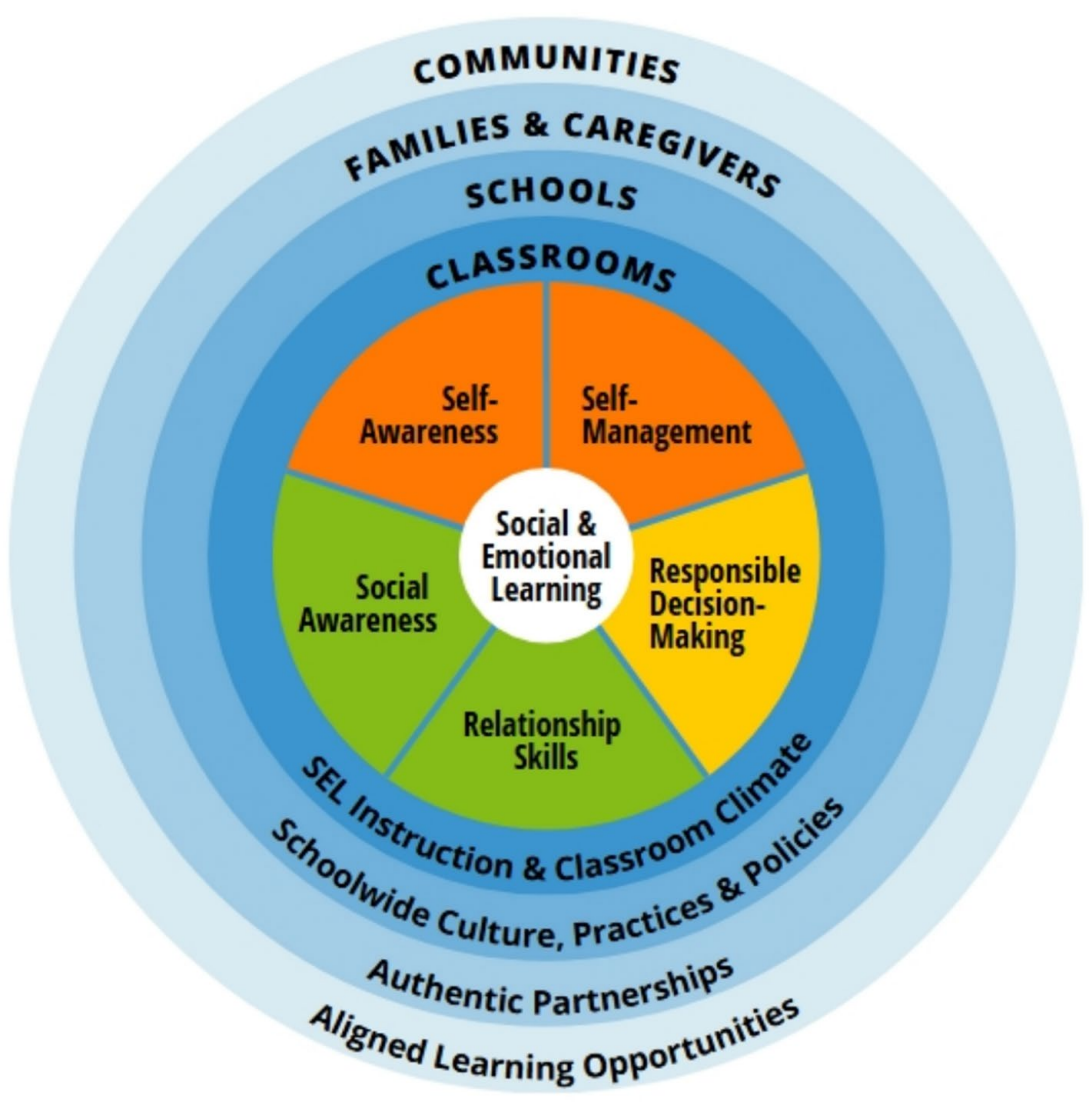
Components of social and emotional learning framework (Collaborative for Academic, Social, and Emotional Learning, 2021).

SEL was a key component, with activities integrated around five core competencies: self-awareness, self-management, social awareness, relationship skills, and responsible decision-making. These competencies were woven into game activities, fostering emotional development and cultural understanding among participants. A PowerPoint presentation highlighted these five key competencies, as illustrated in Figure 1 (Collaborative for Academic, Social, and Emotional Learning, 2021), showing the contents and interrelationships of each competency. This presentation served as a foundation for incorporating SEL principles into various activities and discussions throughout the lessons. The SEL principle was integrated into all aspects of the teaching process during the eight-week course. More specifically, participants’ self-awareness was reflected in their need to reflect on any impressions they may have of American families or educational practices in the first week. During challenging cultural puzzles, instructors encouraged participants to recognize and manage their frustration or confusion when encountering unfamiliar cultural practices. Participants practiced self-management when their initial cultural interpretations proved incorrect. Role-playing activities in Gather.town platform specifically required participants to embody perspectives of individuals from the target culture. For instance, in week 2 (Japan culture), bilingual kindergarten teachers role-played a Japanese parent expressing concerns about their child’s education, helping them practice social awareness. Small group discussions after each treasure hunt emphasized collaborative problem-solving and respectful communication across cultural differences, which enhanced their relationship skills.

##### 3.2.1.4 Gather.town platform

**Figure 2:**
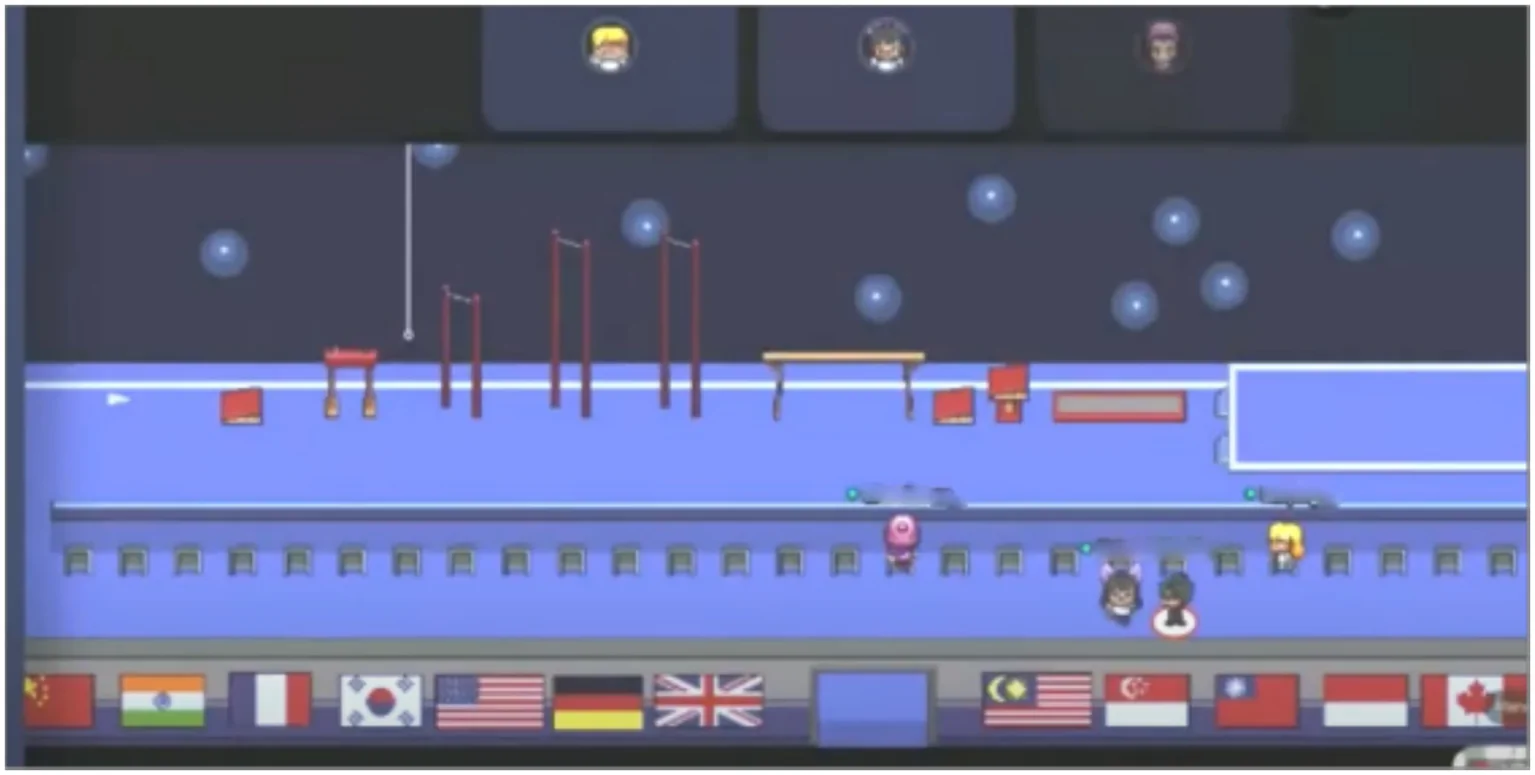
Treasure hunt activity in Gather.town.

The Gather.town platform acted as a virtual environment that facilitated interactive learning experiences for participants. It allowed bilingual kindergarten teachers to engage in cultural detective games, role-playing activities, and small group discussions. Throughout these sessions, bilingual kindergarten teachers collaborated and communicated while actively interacting with the LESCANT Model and SEL concepts. As illustrated in Figure 2, the virtual environment was designed as culturally-themed maps with multiple interconnected rooms, each representing different aspects of the target culture. For example, in Week 1 (USA Culture), the map included: a virtual American suburban home showing family living spaces, an American classroom environment, a community center with various social spaces, a workplace setting demonstrating professional culture, natural environment areas showing relationships with nature. Participants’ avatars could move freely through these spaces, with spatial audio allowing them to hear conversations only when near other participants, simulating real-world cultural immersion. Bilingual kindergarten teachers, organized into small groups, navigate through digital quests designed to explore the cultures and traditions of several nations, such as the USA.

During these treasure hunt activities, bilingual kindergarten teachers encountered clues, puzzles, and challenges related to the LESCANT components, Language, Environment, Social Organization, Context, Authority, Nonverbal Communication, and Time. For example, while solving puzzles, bilingual kindergarten teachers integrated their findings from the LESCANT Model into discussions, enhancing their understanding of cultural differences and similarities in real-time. Each treasure hunt followed a progressive structure. More specifically, in discovery phase, groups explored the virtual environment, collecting cultural clues through observation and interaction. In challenge phase, groups solved progressive puzzles requiring application of cultural understanding.

#### 3.2.2 Instructional materials for the control group

##### 3.2.2.1 Lecture notes

These resources provided information on various cultural aspects of the countries being studied, including history, customs, and traditions, serving as the basis for in-class lectures and discussions. During each weekly session, instructors delivered lectures on these cultural aspects, helping bilingual kindergarten teachers gain an understanding of the countries studied. Lectures included basic cultural facts and explored the reasons behind cultural phenomena, facilitating students’ comprehension of cultural issues.

##### 3.2.2.2 Reading materials

Bilingual kindergarten teachers in the control group were assigned a selection of texts that offer further insights into the cultures being examined. These materials included articles, excerpts, and case studies that complement the content covered in lectures. This allowed bilingual kindergarten teachers to engage in cultural topics at a deeper level outside of class, enhancing their understanding and reflection.

##### 3.2.2.3 Comprehension questions

Instructors designed a set of questions to ensure that bilingual kindergarten teachers understand and could apply the knowledge gained from lectures and reading. These questions encouraged critical thinking and facilitated in-depth discussions regarding the cultural themes addressed during lectures. By answering these questions, bilingual kindergarten teachers engaged with the material and deepened their comprehension.

### 3.3 Measures

#### 3.3.1 Cultural sensitivity questionnaire

The Cultural Sensitivity Questionnaire (CSQ) is a self-report instrument designed to assess individuals’ cultural sensitivity. The original version, developed by Rew et al. (2003), consists of 15 items focused on nurses’ cultural sensitivity. To better align with the context of this study, the questionnaire was revised to include 10 items by omitting those deemed irrelevant, such as “I can adapt to others’ cultural backgrounds during patient interactions.” These modifications ensured that the questionnaire content accurately reflects the experiences of bilingual preschool teachers in China. The final version included impactful and neutral questions, such as, “I am able to communicate effectively with people from different cultures.” Responses were measured on a 5-point Likert scale, ranging from 1 (Strongly Disagree) to 5 (Strongly Agree). Reliability testing yielded a Cronbach’s alpha of *α* = 0.80, indicating good internal consistency. Details can be seen in Appendix B.

#### 3.3.2 Interpersonal response index

The Interpersonal Response Index (IRI) is designed to assess individuals’ capacity to perceive, understand, and respond empathetically in cross-cultural contexts through 10 items. Originally developed by Davis (1983), the IRI comprised 28 items. However, it was adjusted to 10 items to enhance the relevance for this study. The original IRI aimed to measure different dimensions of empathy and were closely related to Western cultural and medical contexts, which may not accurately reflect the experiences of educators in cross-cultural settings. Therefore, items related to medical contexts, such as “I can sense changes in patients’ emotions,” were removed to ensure that the questionnaire is more suited to the actual needs of educators. The revised IRI demonstrated a reliability score of α = 0.84. Reducing the questionnaire to 10 items not only enhanced its cultural applicability but also increased participants’ willingness to complete it, making it more practical for real-world application. Details can be seen in Appendix C.

#### 3.3.3 Intercultural empathy observation checklist

The Intercultural Empathy Observation Checklist (IEOC) is designed to assess key components of intercultural competence by evaluating empathetic behaviors during interactions with individuals from diverse cultural backgrounds. This checklist was based on the framework proposed by Matveev and Merz (2014) in their study on intercultural competence assessment. In this study, the IEOC was rated by two educators and focused on understanding participants’ willingness to empathize with others and engage with different perspectives, examining dimensions such as cognitive understanding of cultural differences, affective cultural empathy, and behavioral demonstrations of empathy. By utilizing a 5-point Likert scale for rating, the IEOC helped researchers assess the effectiveness of interventions aimed at enhancing empathy in cross-cultural contexts. Details can be seen in Appendix D.

#### 3.3.4 Cultural sensitivity observation checklist

The Cultural Sensitivity Observation Checklist (CSOC) evaluates participants’ sensitivity to cultural differences and their ability to adapt their behaviors in cross-cultural interactions. This tool was also based on the concepts outlined by Matveev and Merz (2014) in their framework for assessing intercultural competence, focusing on participants’ cognitive awareness of cultural norms, emotional sensitivity to the feelings and experiences of others, and behavioral adaptability in diverse cultural settings. By employing a structured 5-point Likert scale for evaluation, the CSOC which rated by two raters offered a systematic way to gauge cultural sensitivity. Details can be seen in Appendix E.

#### 3.3.5 Semi-structured interviews

Semi-structured interviews were conducted with a subset of 20 participants (10 from each group) to explore their experiences and perceptions related to cultural sensitivity and intercultural empathy. This interview, consisting of 10 questions, aimed to provide deeper insights into the interventions’ effectiveness while revealing participants’ challenges in cross-cultural interactions during the learning process. These findings had the potential to supplement and extend quantitative data. Details can be seen in Appendix F.

### 3.4 Data analysis

A mixed-methods approach was employed, incorporating both quantitative and qualitative analyses for a comprehensive understanding of the interventions’ effectiveness in enhancing cultural sensitivity and intercultural empathy. Before the main data collection, a one-week pilot study involving 20 participants was conducted to validate the effectiveness and comprehensibility of the questionnaires and observation tools. The purpose of this preliminary research was to identify and revise unsuitable items based on participant feedback regarding the appropriateness and clarity of the questionnaires. Specifically, this feedback prompted us to modify the CSQ and the IRI. For specific reliability statistics, please refer to Table 1.

**Table 1:** Reliability statistics.

Scale	Cronbach’s alpha	*N* of items
CSQ	0.805	10
IRI	0.842	10

When it comes to quantitative analysis, data was analyzed using SPSS 28.0 software. Reliability and intraclass correlation coefficient (ICC) analyses ensured the questionnaires and observation checklists’ reliability and consistency. Independent sample t-tests and paired sample t-tests compared the pretest and posttest scores of both groups. Pearson correlation coefficients examined the relationship between the CSQ, IRI, IEOC, and CSOC scores. In terms of qualitative analysis, thematic analysis, following Braun and Clarke's (2006) guidelines, was applied. Transcripts were read and re-read, with initial codes generated based on recurring patterns and themes related to participants’ experiences and perceptions of cultural sensitivity and intercultural empathy. These codes were organized into broader themes, which were reviewed and refined to ensure their coherence and relevance to the research question. Finally, the themes were defined and named, and illustrative quotes were selected to provide evidence and enrich the interpretation of quantitative findings.

### 3.5 Ethical considerations

This study has obtained ethical approval from Taylor’s University with identity card number 0345085. Participants provided informed consent before taking part in the study, and all data was anonymized to protect participants’ privacy. The researchers ensured that the confidentiality of the participants’ information was maintained throughout the study, and no identifying information was disclosed in any reports or publications.

## 4 Results

### 4.1 Quantitative findings

The reliability statistics in Table 1 demonstrate good reliability for both the CSQ and IRI scales, with Cronbach’s Alpha coefficients above 0.8. This level of reliability signifies that the questionnaire items are appropriately designed, and the data collected are highly reliable for further analysis. The ICC values in Table 2 show that the IEOC and CSOC scores have good internal consistency, both exceeding 0.7. This indicates credible and consistent ratings from the two independent raters, making the checklists suitable for further analysis.

**Table 2:** Intraclass correlation coefficient.

Checklist	ICC	*p*
IEOC	0.771	0.000
CSOC	0.712	0.000

The pre-test scores of the two groups are compared in Table 3, revealing no significant differences between the experimental and control groups in both CSQ and IRI scores. This result indicates that the initial scores of the two groups are comparable and suitable for comparative experiments. The post-test comparison also in Table 3 reveals that the experimental group had significantly higher CSQ and IRI scores than the control group. This finding suggests that playing digital culture detective games in the experimental group resulted in better outcomes in terms of cultural sensitivity and empathy compared to participating in a traditional cross-cultural course in the control group.

**Table 3:** Pre and post test comparison between experimental and control groups.

Scale		Experimental group (*n* = 30)	Control group (*n* = 30)	*t*	*p*	Cohen’s *d*
Pre-test	CSQ	3.10 ± 0.46	3.18 ± 0.56	−0.581	0.564	0.150
	IRI	3.18 ± 0.61	3.29 ± 0.52	−0.756	0.453	0.195
Post-test	CSQ	3.64 ± 0.53	3.37 ± 0.53	2.015	0.049	0.520
	IRI	3.68 ± 0.48	3.39 ± 0.51	2.264	0.027	0.584

In Table 4, the participants in experimental group show significant increases in both CSQ and IRI scores from the pre-test to post-test. This outcome indicates that playing digital culture detective games had a positive impact on their cultural sensitivity and empathy, with medium to large effect sizes (Cohen’s *d*). The participants in control group also show significant increases in both CSQ and IRI scores from the pretest to posttest. However, the improvements are smaller compared to the experimental group, suggesting that the traditional cross-cultural course was less effective than the digital culture detective games in improving cultural sensitivity and empathy, with small to medium effect sizes (Cohen’s *d*).

**Table 4:** Comparison of Pre and Post Scores.

Scale		Pre-test	Post-test	*t*	*p*	Cohen’s *d*
**Experimental group**	CSQ	3.10 ± 0.46	3.64 ± 0.53	−4.624	0.000	0.844
	IRI	3.18 ± 0.61	3.68 ± 0.48	−3.784	0.000	0.691
**Control group**	CSQ	3.18 ± 0.56	3.37 ± 0.53	−2.148	0.040	0.392
	IRI	3.29 ± 0.52	3.39 ± 0.51	−2.684	0.012	0.490

In Table 5, the experimental group has significantly higher IEOC and CSOC scores compared to the control group, supporting the notion that digital culture detective games were more effective in improving intercultural effectiveness and cultural sensitivity. The effect sizes (Cohen’s *d*) indicate medium to large differences between the groups. Table 6 presents significant positive correlations between the CSQ and IRI scores with the IEOC and CSOC scores.

**Table 5:** Comparison of ratings between two groups of participant.

Checklist	Experimental group (*n* = 30)	Control group (*n* = 30)	*t*	*p*	Cohen’s *d*
IEOC	3.66 ± 0.52	3.31 ± 0.60	2.378	0.021	0.614
CSOC	3.82 ± 0.48	3.23 ± 0.46	4.815	0.000	1.243

**Table 6:** Correlation analysis.

Checklist	Index	CSQ	IRI
IEOC	Correlation coefficient	0.875	0.911
	*p*	0.000	0.000
CSOC	Correlation coefficient	0.636	0.571
	*p*	0.000	0.000

### 4.2 Qualitative findings

Table 7 provides a visual representation of the main themes identified through the thematic analysis of the interview data and highlights the differences in the experiences and perceptions of participants in the experimental and control groups. The table gives a comprehensive overview of the differences in experiences and perceptions between the two groups, demonstrating the impact of the interventions on participants’ cultural sensitivity and intercultural empathy, as well as the perceived benefits and limitations of each approach.

**Table 7:** Themes and quotes.

Theme	Quote (Experimental Group)	Quote (Control Group)
Increased cultural awareness	“The treasure hunt game on Gather.town made me realize that there are so many cultural nuances I wasn’t aware of before. It also integrated SEL principles by encouraging self-awareness.” (Participant E3)	“I learned a lot about other cultures during the lectures, but it wasn’t as immersive. I felt that interactive activities could be helpful.” (Participant C2)
Enhanced empathy and perspective-taking	“The role-playing activities on Gather.town helped me understand what people from other cultures might be going through and how they feel. It promoted empathy and social awareness from the SEL framework.” (Participant E7)	“I tried to understand others’ perspectives, but it was more challenging without a hands-on experience. I wish we could have tried some interactive activities.” (Participant C5)
Improved communication skills	“I feel more confident in my ability to communicate with people from different cultural backgrounds after playing the game on Gather.town. The games also helped develop relationship skills from the SEL framework.” (Participant E4)	“I think I improved my communication skills through the discussions, but some practical exercises might have been beneficial. The lectures and readings could be a bit boring.” (Participant C9)
Greater appreciation for diversity	“The cultural puzzles game on Gather.town made me appreciate the richness and beauty of cultural diversity. We can learn so much from one another. The games integrated the SEL principle of responsible decision-making.” (Participant E1)	“I began to appreciate cultural diversity more, but I still feel like I have a lot to learn. I think interactive activities like role-playing could offer a richer learning experience.” (Participant C7)
Enjoyment and engagement	“I looked forward to playing the game on Gather.town every week. It was fun, and I learned a lot without even realizing it. It engaged us emotionally and socially, which aligns with the SEL framework.” (Participant E9)	“The lectures were informative, but I think I would have enjoyed a more interactive approach. The reading assignments could be monotonous at times.” (Participant C3)

#### 4.2.1 Increased cultural awareness

The theme of increased cultural awareness illustrates how the digital cultural detective games in the experimental group, facilitated through the Gather.town platform, provided an immersive experience that helped participants recognize the intricacies of various cultures. These games integrated SEL principles, further enriching the learning experience. In contrast, control group participants acknowledged learning about other cultures through traditional lectures but felt the experience was less immersive and expressed a desire for more interactive activities.

#### 4.2.2 Enhanced empathy and perspective-taking

Enhanced empathy and perspective-taking emerged as another theme, highlighting the ability of digital cultural detective games to facilitate a deeper understanding of the feelings and experiences of people from different cultural backgrounds, while also integrating SEL principles such as social awareness. Control group participants reported more difficulty in achieving the same level of empathy and perspective-taking without hands-on experience.

#### 4.2.3 Improved communication skills

Participants in the experimental group expressed increased confidence in their ability to communicate with individuals from diverse cultural backgrounds after engaging in the games, which also helped develop relationship skills from the SEL framework. Control group participants, while recognizing some improvement in their communication skills, expressed a desire for more practical exercises to further enhance these abilities.

#### 4.2.4 Greater appreciation for diversity

The theme of greater appreciation for diversity indicates that the digital cultural detective games helped experimental group participants develop a deeper admiration for the richness and beauty of various cultures while integrating the SEL principle of responsible decision-making. Although control group participants also reported an increased appreciation for cultural diversity, they felt they still had much to learn in this area and suggested that interactive activities like role-playing could offer a richer learning experience.

#### 4.2.5 Enjoyment and engagement

Participants found digital cultural detective games to be enjoyable and engaging, allowing them to learn without consciously realizing it. These games also engaged them emotionally and socially, aligning with the SEL framework. In comparison, control group participants found traditional lectures informative but expressed a preference for a more interactive and engaging approach, mentioning that the reading assignments could be monotonous at times.

## 5 Discussion

Current study explored the integration of digital cultural detective games with the SEL framework in cultural training programs for kindergarten teachers in China, focusing on the enhancement of cultural sensitivity (RQ1) and intercultural empathy (RQ2). The findings indicate that the experimental group had significant improvements in both the cultural sensitivity and the interpersonal reactivity, suggesting that the integration of game-based learning can effectively help participants understand and respect diverse cultural backgrounds. This aligns with previous research by Anderton and King (2016) and Yang and Kang (2022), supporting the effectiveness of game-based learning in enhancing educators’ intercultural competence. Participants engaged in digital cultural detective games gained a deeper understanding of cultural differences, which made them more sensitive in their actual teaching practices. Regarding intercultural empathy, the enhancements in the experimental group’s IRI scores underscore the effectiveness of digital cultural detective games in enhancing their empathy. This is crucial, as empathy plays a key role in establishing connections with others and has a profound impact on their social and emotional development.

When addressing how kindergarten teachers perceive the impact of participating in digital cultural detective games on their cultural sensitivity and intercultural empathy (RQ3), qualitative data analysis revealed that participants in the experimental group reported a marked increase in cultural awareness, empathy, and communication skills. One participant mentioned that through the games conducted on the Gather.town platform, he gained a deeper understanding of cultural differences, emphasizing the value of immersive experiences in the educational process. Others also noted that the interactive learning environment motivated them to engage in meaningful exchanges within multicultural settings. These findings are consistent with existing literature on game-based learning and interactive learning (Chen and Fan, 2025a; Chen and Fan, 2025b; Dahya, 2009), indicating that this model can promote critical thinking and collaborative abilities.

## 6 Limitations

This study has several limitations. First, the relatively small sample size may affect the generalizability of the findings. Second, modifications to the original standardized questionnaires necessitate that future researchers conduct long-term studies to validate their effectiveness and reliability. Third, due to the focus of this study on kindergarten teachers, who are adults with clear learning goals, they are less likely to be distracted by games during the learning process. If the results are replicated to other populations, such as young children or adolescents, they may have different learning motivations and typically prefer to explore and enjoy fun through games, which increases the risk of potential interference in the educational environment. Games designed for young children or adolescents must ensure that educational objectives align with entertainment value to effectively facilitate learning. Future research could explore the effectiveness of digital cultural detective games in various educational contexts and populations, as well as their long-term impact on kindergarten teachers’ cultural sensitivity and intercultural empathy. Additionally, studies should analyze specific game design elements and instructional strategies to provide insights into the development of more effective educational games.

## 7 Conclusion

This study investigated the integration of digital cultural detective games into social–emotional learning on Gather.town platform to enhance cultural sensitivity and intercultural empathy among kindergarten teachers. The results indicated that the experimental group engaged in digital games exhibited significantly improved cultural sensitivity and intercultural empathy compared to the control group. Research has theoretical and practical significance. On the one hand, this study contributes to the theoretical understanding of how cultural sensitivity and intercultural empathy can be developed through interactive and engaging methods. It highlights the importance of incorporating technology into SEL and expands the existing SEL frameworks by integrating digital cultural detective games, which can serve as a model for future research. The mixed-methods approach utilized also adds depth to the research on educational interventions. On the other hand, the study’s findings can inform the design of teacher training programs by integrating digital cultural detective games into curricula. This also enhances the professional development of kindergarten teachers, making them better equipped to handle diverse classrooms. The identified themes from the qualitative findings can be used to provide specific strategies for educators. For instance, understanding the benefits and limitations of various intervention measures allows teachers to select appropriate methods tailored to their students’ needs. Policymakers may be encouraged to support the integration of technology in teacher training and to prioritize cultural competency in educational standards.

## Data Availability

The original contributions presented in the study are included in the article/Supplementary material, further inquiries can be directed to the corresponding author/s.
