# Correction: Integrating digital cultural detective games with social emotional learning to foster cultural sensitivity and intercultural empathy among kindergarten teachers: a mixed-methods study

**DOI:** 10.3389/fpsyg.2026.1965586

**Published:** 2026-09-16

**Authors:** Guan Zheng Chen, Yuying Fan, Zhongyuan Liao

**Affiliations:** 1 School of Foreign Languages, Guangzhou Xinhua University, Guangzhou, China; 2 School of Public Administration, Guangzhou Xinhua University, Guangzhou, China; 3 School of Teacher Education, Hezhou University, Hezhou, China

**Keywords:** cultural sensitivity, digital cultural detective game, game-based learning, intercultural empathy, kindergarten teachers, social–emotional learning

The reference for the LESCANT Model was omitted. It should read:

Victor, D. A. (1992). *International Business Communication*. New York, NY: HarperCollins Publishers.

Victor (1992) was not cited in the article. The citation has now been inserted in section **3.2 Research design**, *sub-section 3.2.1.1 Digital cultural detective games*, second paragraph and should read: “During the game, bilingual kindergarten teachers took on the role of “cultural detectives,” exploring and solving puzzles based on provided clues. They applied critical thinking to analyze the clues and engage in discussions with peers, sharing varying insights and interpretations. Bilingual kindergarten teachers also used the LESCANT Model Worksheet to record their observations and reflections, assisting them in systematically analyzing various cultural dimensions (Victor, 1992). After gameplay, the instructor facilitated a reflection discussion, encouraging bilingual kindergarten teachers to share their findings which enhance their understanding of cultural knowledge.”, and also in *sub-section 3.2.1.2 LESCANT model worksheet*, second paragraph and should read: “The worksheet provided overviews for each cultural dimension, along with guiding questions and sections for note-taking. Details can be seen in Appendix G. As bilingual kindergarten teachers participated in the treasure hunts, they documented their observations and insights, facilitating deeper explorations of cultural concepts. The structured format of the LESCANT Model Worksheet (Victor, 1992) not only supported bilingual kindergarten teachers in systematically analyzing different cultural dimensions but also encouraged group discussions and reflective debriefing sessions afterward.”

The original version of this article has been updated.

