# Supplementary material for: Integrating digital cultural detective games with social emotional learning to foster cultural sensitivity and intercultural empathy among kindergarten teachers: a mixed-methods study
Source: Front Psychol. 2026 Jan 15;16:1717522. doi: 10.3389/fpsyg.2025.1717522 (PMC12853197; doi:10.3389/fpsyg.2025.1717522)
Supplement: Supplementary file 1 [file Supplementary_file_1.docx]

**Appendices**

Appendix A. Lesson Plan for Experimental and Control Groups

| **Week** | **Topic** | **Experimental Group Lesson Plan** | **Control Group Lesson Plan** |
| --- | --- | --- | --- |
| 1 | Introduction to Cultural Awareness and USA Culture | (10 mins) Introduce Gather.town and integration of cultural detective methods with social emotional learning principles  (20 mins) Presentations on USA Culture  (30 mins) Treasure Hunt and Cultural Puzzles on USA culture  (20 mins) Role-playing activities and small group discussion about cultural experiences  (10 mins) Wrap up and reflection | (10 mins) Introduce the outline of the cultural lesson  (20 mins) Lecturing on USA Culture  (30 mins) Reading and comprehension questions on USA culture  (20 mins) Small group discussion about cultural experiences  (10 mins) Wrap up and reflection |
| 2 | Exploring Japan Culture | (10 mins) Icebreaker: Cultural Bingo  (5 mins) Recap cultural detective methods and principles of social emotional learning  (20 mins) Presentation on Japan culture  (30 mins) Treasure Hunt and Cultural Puzzles on Japan culture  (20 mins) Role-playing activities and small group discussion about cultural experiences  (5 mins) Wrap up and reflection | (10 mins) Icebreaker: Cultural Bingo  (5 mins) Recap of last lesson  (20 mins) Lecturing on Japan culture  (30 mins) Reading and comprehension questions on Japan culture  (20 mins) Small group discussion about cultural experiences  (5 mins) Wrap up and reflection |
| 3 | Understanding China Culture | (10 mins) Icebreaker: Common Ground  (5 mins) Recap cultural detective methods and principles of social emotional learning  (20 mins) Presentation on China culture  (30 mins) Treasure Hunt and Cultural Puzzles on China culture  (20 mins) Role-playing activities and small group discussion about cultural experiences  (5 mins) Wrap up and reflection | (10 mins) Icebreaker: Common Ground  (5 mins) Recap of last lesson  (20 mins) Lecturing on China culture  (30 mins) Reading and comprehension questions on China culture  (20 mins) Small group discussion about cultural experiences  (5mins) Wrap up and reflection |
| 4 | Exploring India Culture | (10 mins) Icebreaker: Would You Rather?  (5 mins) Recap cultural detective methods and principles of social emotional learning  (20 mins) Presentation on India culture  (30 mins) Treasure Hunt and Cultural Puzzles on India culture  (20 mins) Role-playing activities and small group discussion about cultural experiences  (5 mins) Wrap up and reflection | (10 mins) Icebreaker: Would You Rather?  (5 mins) Recap of last lesson  (20 mins) Lecturing on India culture  (30 mins) Reading and comprehension questions on India culture  (20 mins) Small group discussion about cultural experiences  (5mins) Wrap up and reflection |
| 5 | Exploring Brazil Culture | (10 mins) Icebreaker: Cross-Cultural Scavenger Hunt  (5 mins) Recap cultural detective methods and principles of social emotional learning  (20 mins) Presentation on Brazil culture  (30 mins) Treasure Hunt and Cultural Puzzles on Brazil culture  (20 mins) Role-playing activities and small group discussion about cultural experiences  (5 mins) Wrap up and reflection | (10 mins) Icebreaker: Cross-Cultural Scavenger Hunt  (5 mins) Recap of last lesson  (20 mins) Lecturing on Brazil culture  (30 mins) Reading and comprehension questions on Brazil culture  (20 mins) Small group discussion about cultural experiences  (5 mins) Wrap up and reflection |
| 6 | Exploring South Africa Culture | (10 mins) Icebreaker: Cultural Charades  (5 mins) Recap cultural detective methods and principles of social emotional learning  (20 mins) Presentation on South Africa culture  (30 mins) Treasure Hunt and Cultural Puzzles on South Africa culture  (20 mins) Role-playing activities and small group discussion about cultural experiences  (5 mins) Wrap up and reflection | (10 mins) Icebreaker: Cultural Charades  (5 mins) Recap of last lesson  (20 mins) Lecturing on South Africa culture  (30 mins) Reading and comprehension questions on South Africa culture  (20 mins) Small group discussion about cultural experiences  (5 mins) Wrap up and reflection |
| 7 | Exploring Russia Culture | (10 mins) Icebreaker: Two Truths and a Lie  (5 mins) Recap cultural detective methods and principles of social emotional learning  (20 mins) Presentation on Russia culture  (30 mins) Treasure Hunt and Cultural Puzzles on Russia culture  (20 mins) Role-playing activities and small group discussion about cultural experiences  (5 mins) Wrap up and reflection | (10 mins) Icebreaker: Two Truths and a Lie  (5 mins) Recap of last lesson  (20 mins) Lecturing on Russia culture  (30 mins) Reading and comprehension questions on Russia culture  (20 mins) Small group discussion about cultural experiences  (5 mins) Wrap up and reflection |
| 8 | Exploring Australia Culture | (10 mins) Icebreaker: Cultural Trivia  (5 mins) Recap cultural detective methods and principles of social emotional learning  (20 mins) Presentation on Australia culture  (30 mins) Treasure Hunt and Cultural Puzzles on Australia culture  (20 mins) Role-playing activities and small group discussion about cultural experiences  (5 mins) Wrap up and reflection | (10 mins) Icebreaker: Cultural Trivia (5 mins) Recap of last lesson  (20 mins) Lecturing on Australia culture  (30 mins) Reading and comprehension questions on Australia culture  (20 mins) Small group discussion about cultural experiences  (5 mins) Wrap up and reflection |

Appendix B. Cultural Sensitivity Questionnaire (CSQ)

| **Demographic Information** |  |
| --- | --- |
| 1. Age |  |
| 2. Gender |  |
| 3. Ethnicity |  |
| 4. Nationality |  |
| 5. Language(s) spoken |  |

| **Cultural Sensitivity Questionnaire** | **Strongly Disagree** | **Disagree** | **Neither Agree nor Disagree** | **Agree** | **Strongly Agree** |
| --- | --- | --- | --- | --- | --- |
| 1. I feel comfortable interacting with people from different cultures. |  |  |  |  |  |
| 2. I can adapt my behavior to fit in with people from different cultures. |  |  |  |  |  |
| 3. I enjoy learning about different cultures. |  |  |  |  |  |
| 4. I am aware of my own cultural values and biases. |  |  |  |  |  |
| 5. I am able to recognize when I am making cultural mistakes. |  |  |  |  |  |
| 6. I am able to understand cultural differences. |  |  |  |  |  |
| 7. I am able to respect cultural differences. |  |  |  |  |  |
| 8. I am willing to adapt my behavior to accommodate cultural differences. |  |  |  |  |  |
| 9. I am able to work effectively in culturally diverse teams. |  |  |  |  |  |
| 10. I am able to communicate effectively with people from different cultures. |  |  |  |  |  |

Appendix C. Davis Interpersonal Response Index (IRI)

| **Demographic Information** |  |
| --- | --- |
| 1. Age |  |
| 2. Gender |  |
| 3. Ethnicity |  |
| 4. Nationality |  |
| 5. Language(s) spoken |  |

| **IRI Question** | **Strongly Disagree** | **Disagree** | **Neither Agree nor Disagree** | **Agree** | **Strongly Agree** |
| --- | --- | --- | --- | --- | --- |
| 1. I often have tender, concerned feelings for people from other cultures. | 1 | 2 | 3 | 4 | 5 |
| 2. I sometimes try to understand people from other cultures better by imagining how things look from their perspective. | 1 | 2 | 3 | 4 | 5 |
| 3. When I see someone from another culture being taken advantage of, I feel kind of protective towards them. | 1 | 2 | 3 | 4 | 5 |
| 4. I sometimes feel overwhelmed by the emotions of people from other cultures. | 1 | 2 | 3 | 4 | 5 |
| 5. I believe that there are two sides to every question and try to look at them both, even in cross-cultural situations. | 1 | 2 | 3 | 4 | 5 |
| 6. I often have difficulty understanding abstract cultural concepts or values. | 1 | 2 | 3 | 4 | 5 |
| 7. It upsets me to see animals or people from other cultures suffering. | 1 | 2 | 3 | 4 | 5 |
| 8. I try to look at everybody's side of a disagreement before I make a decision, even in cross-cultural situations. | 1 | 2 | 3 | 4 | 5 |
| 9. I sometimes feel like a "psychic sponge" that absorbs the emotions of people from other cultures around me. | 1 | 2 | 3 | 4 | 5 |
| 10. Becoming extremely involved in a good book, movie, or other cultural media is somewhat rare for me. | 1 | 2 | 3 | 4 | 5 |

Appendix D. Intercultural Empathy Observation Checklist (IEOC)

Observer: _____________________________ Date: _______________

Group: _______________

Instructions: Observe the participant during the treatment session and rate the following items using a 5-point Likert scale (1 = Strongly Disagree, 2 = Disagree, 3 = Neither Agree nor Disagree, 4 = Agree, 5 = Strongly Agree).

| **Observation Items** | **Rating** |
| --- | --- |
| Demonstrates willingness to understand others' perspectives, even if different from their own. |  |
| Demonstrates sensitivity to others' emotions and cultural background. |  |
| Uses appropriate language and tone when communicating with individuals from different cultures. |  |
| Displays openness to different cultures, perspectives, and beliefs. |  |
| Takes active steps to build rapport with people from different cultures. |  |
| Demonstrates willingness to help others, regardless of cultural background. |  |
| Is able to identify and articulate others' feelings and experiences, even if different from their own. |  |
| Demonstrates patience and understanding towards others, especially when faced with cultural differences. |  |
| Shows respect for others' cultural beliefs, practices, and traditions. |  |
| Actively listens to others and tries to understand their experiences and cultural background. |  |
| Total Score: _______ |  |
| Interpretation: The Intercultural Empathy Observation Checklist (IEOC) is a tool designed to measure a participant's intercultural empathy. Higher scores indicate greater levels of intercultural empathy. The IEOC may be useful in assessing the impact of interventions on intercultural empathy. | |

Appendix E. Cultural Sensitivity Observation Checklist (CSOC)

Observer: _____________________________ Date: _______________

Group: _______________

Instructions: Observe the participant during the treatment session and rate the following items using a 5-point Likert scale (1 = Strongly Disagree, 2 = Disagree, 3 = Neither Agree nor Disagree, 4 = Agree, 5 = Strongly Agree).

| \| **Observation Items** \| **Rating** \| \| --- \| --- \| \| Demonstrates respect for different cultural practices \|  \| \| Shows interest in learning about other cultures \|  \| \| Avoids making assumptions about other cultures \|  \| \| Uses appropriate language and tone when discussing culture \|  \| \| Displays sensitivity to cultural differences \|  \| \| Actively listens to others when discussing culture \|  \| \| Acknowledges own cultural biases and limitations \|  \| \| Adapts communication style to meet cultural needs \|  \| \| Expresses curiosity about cultural similarities \|  \| \| Responds appropriately to cross-cultural misunderstandings \|  \| \| Total Score: _______ \| \|   Interpretation: Higher scores indicate greater levels of cultural sensitivity. This observation checklist was used to assess the participant's level of cultural sensitivity during the treatment session. The data collected was analyzed using descriptive statistics to provide an overall summary of the participant's cultural sensitivity. |
| --- | --- | --- | --- | --- | --- | --- | --- | --- | --- | --- | --- | --- | --- | --- | --- | --- | --- | --- | --- | --- | --- | --- | --- | --- |

Appendix F. Interview Questions

1. How did the Gather.town-based Cultural Communication Training Program (or Traditional Cross-Cultural Course) impact your understanding of different cultures?

2. In what ways did the program (or course) influence your empathy and ability to connect with individuals from diverse cultural backgrounds?

3. Can you describe a specific activity or lesson from the program (or course) that was particularly impactful for your intercultural communication skills?

4. How do you think your experience with the Gather.town platform (or traditional course format) contributed to your engagement and enjoyment of the program (or course)?

Were there any challenges or limitations you encountered during the 5. Gather.town-based program (or traditional course)? If so, can you provide an example?

6. How has your participation in the Gather.town-based program (or traditional course) affected your teaching practices and interactions with students from diverse cultural backgrounds?

7. How did the integration of Social and Emotional Learning (SEL) principles in the Gather.town-based program (or their absence in the traditional course) influence your learning experience?

8. Can you share an example of how the program (or course) helped you become more aware of your own cultural biases and assumptions?

How do you plan to apply the knowledge and skills gained from the 9. 9. Gather.town-based program (or traditional course) to your future teaching practices?

10. Based on your experience, what recommendations would you make to improve the Gather.town-based Cultural Communication Training Program (or Traditional Cross-Cultural Course) for future participants?

Appendix G. LESCANT Model Worksheet

The acronym LESCANT stands for Language, Environment, Social Organization, Context, Authority, Nonverbal Communication, and Time Orientation.

Name: ____________________________ Date: ________________________

Instructions: For each component of the LESCANT model, provide a brief description of how it influences cultural sensitivity and intercultural empathy in the context of the digital culture detective game or the regular cross-cultural course.

Language:

1. How does language impact communication and understanding in cross-cultural interactions?
2. What strategies can be employed to overcome language barriers?

Response: _____________________________________________________________

Environment:

1. How do the physical and social environment influence cultural values and behaviors?
2. How can you adapt to different environments in cross-cultural interactions?

Response: _____________________________________________________________

Social Organization:

1. How do social structures, family dynamics, and group affiliations affect cross-cultural interactions?
2. What can you do to show respect and understanding of different social organizations?

Response: _____________________________________________________________

Context:

1. How does the high/low context of a culture influence communication styles and expectations?
2. What strategies can be employed to adapt to different cultural contexts?

Response: _____________________________________________________________

Authority:

1. How do power dynamics and hierarchies impact cross-cultural interactions?
2. How can you navigate and respect different authority structures in diverse cultures?

Response: _____________________________________________________________

Nonverbal Communication:

1. What are some common nonverbal cues that may differ across cultures?
2. How can you improve your nonverbal communication skills to enhance intercultural empathy?

Response: _____________________________________________________________

Time Orientation:

1. How do different cultures perceive and value time, and how does this affect interactions?
2. What can you do to adapt to and respect different time orientations in cross-cultural situations?

Response: _____________________________________________________________

Reflection:

1. What insights have you gained from completing this LESCANT Model Worksheet?
2. How will you apply these insights to enhance your cultural sensitivity and intercultural empathy in future cross-cultural interactions?

Response: _____________________________________________________________

Appendix H. LESCANT Model Worksheet Filled Sample

Name: Participant E7

Date: Week 1 of Intervention

Instructions: For each component of the LESCANT model, provide a brief description of how it influences cultural sensitivity and intercultural empathy in the context of the digital culture detective game or the regular cross-cultural course.

Language:

1. How does language impact communication and understanding in cross-cultural interactions?

2. What strategies can be employed to overcome language barriers?

Response: Americans tend to use direct communication styles, saying exactly what they mean. I noticed phrases like "Let's get straight to the point" and "Can you clarify that?" which show preference for explicit communication rather than indirect hints. In my culture, we often rely on context and non-verbal cues. For example, a mother directly said, "I don't think this approach is working," whereas in my culture, she might say, "Perhaps we could try something different?" To overcome these differences, I learned to: (1) clarify my understanding by restating what I heard, (2) explain to American parents that I value their direct feedback, and (3) practice being more explicit in my own communication while respecting my communication style. Understanding this helps me interpret American parents' feedback as constructive rather than confrontational.

Environment:

3. How does the physical and social environment influence cultural values and behaviors?

4. How can you adapt to different environments in cross-cultural interactions?

Response: The physical classroom setup emphasizes individual workspaces with personal cubbies and name labels, reflecting values of personal ownership and independence. Circle time areas promote face-to-face interaction and equality. Materials are placed at child height for self-selection. Each child has their own labeled storage space, contrasting with shared storage in my home culture. This taught me that environment design communicates cultural values about autonomy and self-directed learning. To adapt, I will: (1) create classroom spaces that balance individual and group needs, (2) provide both personal and shared storage options, (3) arrange furniture to support both independent work and collaborative activities, and (4) use environmental design intentionally to communicate the values I want to promote in my classroom.

Social Organization:

5. How do social structures, family dynamics, and group affiliations affect cross-cultural interactions?

6. What can you do to show respect and understanding of different social organizations?

Response: American early childhood settings emphasize nuclear family structures and individual family units. Parent-teacher conferences focus on the individual child's development without much reference to siblings or extended family. When discussing a child's progress, American teachers rarely ask about family dynamics or older siblings' influence, while in my culture, family context is central to understanding a child. This showed me how individualism extends beyond the child to family interactions. To show respect for different social organizations, I will: (1) ask families about their preferred involvement level, (2) acknowledge that some families prioritize individual achievement while others value family harmony, (3) include options for both individual and family participation in classroom activities, and (4) avoid assumptions about family structures when communicating with parents.

Context:

7. How does the high/low context of a culture influence communication styles and expectations?

8. What strategies can be employed to adapt to different cultural contexts?

Response: High-context vs. low-context communication became apparent in daily routines. American teachers give explicit verbal instructions ("Please wash your hands before snack"), whereas in high-context cultures, children learn through observation and implicit expectations. A teacher said, "Use your words to tell him you're upset," making explicit what might be understood implicitly in my culture. This helps me understand why American children are encouraged to verbalize everything. Strategies to adapt include: (1) being more explicit in my instructions while also teaching children to read contextual cues, (2) explaining the reasoning behind rules rather than expecting implicit understanding, (3) using both verbal and visual cues to support different learning styles, and (4) helping children develop skills in both explicit and implicit communication to prepare them for diverse contexts.

Authority:

9. How do power dynamics and hierarchies impact cross-cultural interactions?

10. How can you navigate and respect different authority structures in diverse cultures?

Response: Power distance is relatively small in American culture. Teachers and students use first names in many settings. Children are encouraged to question and negotiate with adults. A 4-year-old said, "But why do I have to?" when asked to clean up, and the teacher patiently explained rather than simply expecting compliance. This contrasts with the higher power distance in my culture where adult authority is less questioned. Understanding this helps me appreciate the goal of developing independent thinking. To navigate different authority structures, I will: (1) encourage American children to share their opinions while teaching respectful disagreement skills, (2) explain to parents from high power distance cultures that questioning is viewed as engagement rather than disrespect in American settings, (3) establish clear expectations about when children can negotiate and when immediate compliance is necessary, and (4) model respectful dialogue with authority figures.

Nonverbal Communication:

11. What are some common nonverbal cues that may differ across cultures?

12. How can you improve your nonverbal communication skills to enhance intercultural empathy?

Response: Eye contact during conversations is expected and valued as a sign of attention and respect in American culture. Physical space between speakers is relatively large compared to my culture. Teachers consistently said, "Look at me when I'm talking to you," which in some Asian cultures might be considered disrespectful. Also, teachers maintain arm’s length distance during conversations, while my culture accepts closer proximity. Common differences include: eye contact expectations, personal space boundaries, touching norms, and facial expressions. To improve my nonverbal communication skills, I will: (1) practice maintaining culturally appropriate eye contact with American families while explaining my own cultural norms, (2) observe and respect personal space preferences, (3) be aware that my nonverbal cues may be interpreted differently, and (4) teach children that nonverbal communication varies across cultures and to ask when uncertain.

Time Orientation:

13. How do different cultures perceive and value time, and how does this affect interactions?

14. What can you do to adapt to and respect different time orientations in cross-cultural situations?

Response: American culture is highly time-conscious with strict schedules. Classes start and end precisely on time. The daily schedule is posted and followed rigidly – 9:00 arrival, 9:15 circle time, 10:00 snack, etc. A teacher said, "We need to respect everyone's time" when a parent arrived late. In my culture, time is more flexible and relationships take priority over schedules. This helped me understand American emphasis on efficiency and planning. To adapt to and respect different time orientations, I will: (1) establish clear routines and schedules while building flexibility when relationships require it, (2) communicate schedule expectations clearly to all families, (3) understand that punctuality is valued in American contexts without judging my own cultural approach as inferior, and (4) plan buffer time for relationship-building within the structured schedule.

Reflection:

15. What insights have you gained from completing this LESCANT Model Worksheet?

16. How will you apply these insights to enhance your cultural sensitivity and intercultural empathy in future cross-cultural interactions?

Response: This activity made me realize my own cultural biases and helped me develop more empathy for families from different backgrounds. I gained insights that cultural differences are not right or wrong, but reflect different values and priorities. The treasure hunt experience was transformative in helping me see cultural differences as opportunities rather than obstacles. I will apply these insights by: (1) encouraging American children to share their opinions while teaching respectful disagreement skills, (2) creating classroom spaces that balance individual and group needs, (3) explaining my communication style to American parents and letting them know I value their direct feedback, (4) being more explicit in my instructions while also teaching children to read contextual cues, (5) establishing clear routines and schedules while building flexibility when relationships require it, and (6) continuously reflecting on how my cultural background influences my teaching practices. Most importantly, I will approach cultural differences with curiosity rather than judgment and use them as teaching opportunities for children to develop intercultural competence.

Appendix I: Control Group Instructional Materials Sample

Note: These materials represent the traditional lecture-based approach used in the control group.

**Lecture Notes Sample (Week 1: USA Culture)**

Topic: Understanding American Cultural Values in Early Childhood Education

I. Historical Background

A. Immigration history and cultural diversity

B. Democratic foundations and their influence on education

C. Frontier spirit and self-reliance traditions

II. Core American Values

A. Individualism

Emphasis on personal achievement and independence

Encouragement of self-expression from early childhood

Individual rights and personal choice

B. Equality

Democratic ideals in classroom settings

Minimization of hierarchical relationships

Equal treatment regardless of social background

C. Competition

Performance-based recognition systems

Individual assessment and grading

Achievement motivation

III. Social Organization

A. Family Structure

Nuclear family emphasis

Early independence training

Parent-child relationship patterns

B. Education System

Student-centered learning approach

Active participation expectations

Questioning and critical thinking encouraged

IV. Communication Patterns

A. Direct Communication Style

Explicit verbal expression valued

Open disagreement accepted

"Say what you mean" cultural norm

B. Informality

First-name basis common

Casual dress codes

Relaxed teacher-student interactions

V. Time Orientation

A. Monochronic Time Perspective

Punctuality highly valued

Schedule adherence expected

Sequential task completion

**Reading Material Sample (Week 1: USA Culture)**

Understanding American Cultural Values in Early Childhood Education

American culture profoundly influences early childhood education practices, creating distinct classroom dynamics that may differ significantly from Asian educational traditions. Understanding these cultural values is essential for educators working in cross-cultural contexts.

Individualism vs. Collectivism: American culture emphasizes individualism, prioritizing personal goals over group harmony. In early childhood settings, this manifests through activities that encourage self-expression, personal choice, and individual achievement. Children are taught to voice their opinions, make independent decisions, and develop unique identities. Teachers frequently ask, "What do YOU think?" rather than presenting authoritative answers.

Student-Centered Learning: American classrooms typically follow student-centered approaches where children actively participate in their learning process. Teachers serve as facilitators rather than knowledge transmitters. Children are encouraged to ask questions, challenge ideas, and explore topics based on personal interests. This contrasts with more traditional, teacher-directed approaches common in Asian contexts.

Communication Expectations: Direct communication is highly valued in American culture. Children learn to express thoughts and feelings explicitly, and teachers encourage open dialogue. Disagreement is not considered disrespectful but rather a sign of critical thinking. American educators often interpret silence as lack of understanding rather than respect or contemplation.

Independence Training: From early ages, American children are trained toward independence. Preschoolers are expected to manage personal belongings, make choices about activities, and solve conflicts with minimal adult intervention. Parents and teachers view these skills as essential preparation for future self-reliance.

Equality and Authority: American classrooms reflect democratic values with relatively flat hierarchies. Teachers and students interact informally, often on a first-name basis. Children may question authority figures without fear of punishment, and their opinions are taken seriously regardless of age. This egalitarian approach aims to develop confident, independent thinkers.

Practical Implications: For educators from different cultural backgrounds, understanding these values helps navigate American educational settings effectively. Recognizing that silence may be misinterpreted, that questioning shows engagement rather than disrespect, and that independence is systematically cultivated allows for more successful cross-cultural teaching experiences. However, educators should also recognize that maintaining connections to their own cultural values can enrich American classrooms by introducing alternative perspectives on learning, community, and child development.

**Comprehension Questions Sample**

Factual Recall Questions (1-5)

What are three core American values discussed in the lecture?

List two characteristics of American family structure mentioned in the reading.

What does "monochronic time perspective" mean in American culture?

According to the reading, how do American teachers typically view their role in the classroom?

What communication style is predominantly valued in American culture?

Comprehension Questions (6-9)

Explain why American parents might encourage children to question authority figures.

How does the concept of individualism influence classroom activities in American early childhood education?

Describe the difference between student-centered and teacher-directed learning approaches.

Why might silence in an American classroom be interpreted differently than in Asian classrooms

Application Questions (10-12)

Design a classroom activity that honors both American individualism and Chinese collectivism values.

A Chinese parent complains that their child has become "disrespectful" after attending an American preschool. How would you address this concern?

How could you adapt your teaching style to balance direct and indirect communication approaches?

Critical Thinking Questions (13-15)

The reading mentions that American culture values equality, yet social inequality persists. Discuss this contradiction and its implications for education.

Analyze potential advantages and disadvantages of emphasizing independence training in early childhood.

How might American cultural values in education both benefit and challenge international students?

**Small Group Discussion Prompts**

Activity 1: Cultural Values Comparison (20 minutes)

Objective: Compare American and Chinese cultural values in education

Instructions:

Divide into groups of 4-5 participants

Create a Venn diagram comparing American and Chinese educational values

Identify unique characteristics of each culture and shared values

Present findings to the larger group

Discussion Questions:

Which values overlap between the two cultures?

Where are the most significant differences?

How might these differences impact classroom management?

Activity 2: Case Study Discussion (25 minutes)

Case Scenario:

A Chinese preschool teacher in an American classroom notices that children frequently interrupt her during story time, ask many questions, and sometimes disagree with story interpretations. She feels this behavior is disrespectful, but her American supervisor says it shows "active engagement."

Discussion Questions:

What cultural values create this misunderstanding?

How can the teacher reframe her interpretation of children's behavior?

What strategies could help her adapt while maintaining her teaching identity?

How might she educate her supervisor about Chinese educational values?

Activity 3: Communication Style Role-Play (20 minutes)

Objective: Practice recognizing and adapting to different communication styles

Scenario Pairs:

Direct vs. Indirect: Role-play giving constructive feedback to a parent about their child's behavior using both communication styles

Hierarchical vs. Equal: Role-play a teacher-student interaction using formal and informal approaches

Debrief Questions:

Which style felt more natural to you? Why?

What challenges did you experience with the unfamiliar style?

How can you develop flexibility in communication approaches?

Activity 4: Personal Reflection Sharing (15 minutes)

Objective: Connect learning to personal experience

Individual Reflection (5 minutes):

Write brief responses to:

Which American cultural value is most challenging for you to understand or accept?

Which value resonates with your own beliefs?

What specific strategy will you try in your teaching practice?

Group Sharing (10 minutes):

Share one insight from your reflection

Discuss common themes across group members

Identify support strategies for cultural adaptation
